# Modular network construction using eQTL data: an analysis of computational costs and benefits

**DOI:** 10.3389/fgene.2014.00040

**Published:** 2014-02-26

**Authors:** Yen-Yi Ho, Leslie M. Cope, Giovanni Parmigiani

**Affiliations:** ^1^Division of Biostatistics, School of Public Health, University of MinnesotaMinneapolis, MN, USA; ^2^The Sidney Kimmel Cancer Center, Johns Hopkins School of MedicineBaltimore, MD, USA; ^3^Dana-Farber Cancer Institute and Harvard School of Public HealthBoston, MA, USA

**Keywords:** Bayesian networks, search algorithm, network variable selection, eQTL, chemotherapy resistance

## Abstract

**Background:** In this paper, we consider analytic methods for the integrated analysis of genomic DNA variation and mRNA expression (also named as eQTL data), to discover genetic networks that are associated with a complex trait of interest. Our focus is the systematic evaluation of the trade-off between network size and network search efficiency in the construction of these networks.

**Results:** We developed a modular approach to network construction, building from smaller networks to larger ones, thereby reducing the search space while including more variables in the analysis. The goal is achieving a lower computational cost while maintaining high confidence in the resulting networks. As demonstrated in our simulation results, networks built in this way have low node/edge false discovery rate (FDR) and high edge sensitivity comparing to greedy search. We further demonstrate our method in a data set of cellular responses to two chemotherapeutic agents: docetaxel and 5-fluorouracil (5-FU), and identify biologically plausible networks that might describe resistances to these drugs.

**Conclusion:** In this study, we suggest that guided comprehensive searches for parsimonious networks should be considered as an alternative to greedy network searches.

## 1. Introduction

Beginning with work by Schadt et al. (2005), a number of recent studies combine SNP datasets with transcriptional, metabolomic or other data to develop network models for common diseases that link response to treatment (Chen et al., 2008; Schadt, 2009; Chang and McGeachie, 2011). Schadt describes the principle behind this approach: “In the context of common human diseases, the disease states can be considered emergent properties of molecular networks, as opposed to the core biological processes associated with a disease being driven by responses to changes in a small number of genes” (Schadt, 2009). These methods have proved effective in several practical settings (Pe'er et al., 2001; Mehrabian et al., 2005; Zhu et al., 2007; Chen et al., 2008; Yang et al., 2009) but there are open problems and overcoming the computational difficulties associated with high dimensional data analysis is of particular interest. Approaches commonly used to manage the computational burden include reducing the number of genes by pre-filtering based on gene function or the results of univariate analysis, (Imoto et al., 2003; Li et al., 2005; Chang and McGeachie, 2011), and improving the efficiency of the search for solutions, for instance by using greedy algorithms (Friedman et al., 2000; Yu et al., 2002; Teyssier, 2005).

Most recently, hybrid approaches like the H2PC algorithm (Gasse et al., 2012) combine the greedy hill-climbing step with a constraint-based optimization, although these have not yet been adapted for use on a mixture of continuous and discrete variables, limiting their applicability to networks incorporating several types of genomic data. Others have incorporated transcription-factor, or protein–protein binding information from biological knowledge bases to improve gene network inference. The GRAM algorithm (Bar-Joseph et al., 2003), as well as the approaches by Xu et al. (2004), and Tu et al. (2006) are representative of this strategy. Alternatively, other approaches for studying genetic networks consider only pairwise relationships such as correlation or partial correlations (Zhang and Horvath, 2005; Lasserre et al., 2013). These approaches investigate the association between pairs of genes, and hence do not consider the directionality of an edge.

In this study, we plot a unique course suggested to us by Schadt's use of SNP-transcript-phenotype trios in causal analysis (Schadt et al., 2005), wherein we build the causal network up modularly from smaller, data-driven network components. Here *network* is used in the sense of Bayesian networks, our tool of choice for describing the dependence structure between variables. At the most basic level, this can be thought of as a strategy for selecting the most informative genomic and transcriptomic sites for use in network models. Although they did not incorporate the philosophy into variable selection, Pe'er et al. (2001) also emphasized the value of basing network inferences on small but high-confidence subnetworks: “We hypothesize that if we can find a subnetwork … with a relatively high confidence, then our estimate of edges and other features in this region will be more reliable. While a full-scale network is currently of insufficient quality, statistically significant sub-networks can be reconstructed. Indeed, such subnetworks often correspond to biologically meaningful relations between genes” (Pe'er et al., 2001). The goal is to strike a balance between the high computational costs of large scale network analysis, on the one hand, and the loss of information contained in the data necessitated by aggressive pre-filtering steps and greedy approaches to network development on the other. We are looking for an equilibrium point where component networks are small enough that searching through them is computationally feasible but large enough to capture important network substructures.

We propose a network-driven feature selection strategy, whereby sets of variables are chosen on the basis of their role in small subnetworks, and then iteratively assembled into larger structures. To investigate the utility of this approach, referred to as nPARS for **network Partition and Reassembly Search**, we evaluate it in an extensive set of biologically plausible simulations, comparing it to a gold standard exhaustive search for a best fitting network as well as the commonly-used greedy hill-climbing algorithm. We also demonstrate our proposed approach in a data set of cellular responses to two chemotherapeutic agents: docetaxel and 5-fluorouracil (5-FU) and discuss possible extensions.

## 2. Methods

### 2.1. Bayesian networks for genetic network discovery

We chose Bayesian networks to represent the widely used class of network models that aim to capture the dependence structure in a dataset. A particularly attractive feature of Bayesian networks is their ability to accommodate genomic data of various types by using continuous or discrete nodes to represent variables under consideration, for example: continuous nodes to represent continuous measurements such as gene expression, and discrete nodes to represent discrete variable such as genotype.

Given a Bayesian network structure, the approach to calculate likelihood and network score has been well-established in the literature. The novelty of this paper is to introduce the nPARS search algorithm, described in section 2.2, to guide the search process and to visit parts of the network space that reflect parts of the true underlying network structure in a given data, since the search space is oftentimes enormous. Formally, a Bayesian network is a graphical representation of the joint distribution of a set of variables (Pearl, 1988) consisting of two components: (1) a directed acyclic graph in which nodes correspond to random variables, and directed edges to dependencies between variables; for example, L → E indicates that the status at node L is associated with the alteration of status of node E. And (2) the joint distribution of the random variables decomposed according to the graphical model, under an assumption of Markov conditional independence.

Thus the dependence structure can be described as *P*(*X*_1_, *X_2_, …, *X_p_*|*G*) = ∏^*p*^_*i*_*P* (*X*_i_*|*Pa*(*X*_*i*_), *G*), where *Pa*(*X_i_*) represents the parents of nodes *X_i_* in graph *G*. The conditional distributions in the described equation were specified according to the types (discrete or continuous) of *X_i_*. For discrete nodes, we assume they follow multinomial distribution with parameter θ_*d*_ and the prior distribution of θ_*d*_ follows Dirichlet. For continuous nodes, we assume linear Gaussian conditional densities given the value of its parents and apply Gaussian-inverse gamma priors. For example, assuming a continuous node, *X_i_*, has both continuous parents (*Pa_c_*)and discrete parents (*Pa_d_*), we apply the following distribution model:

$$\begin{array}{l} P\text{​}\left(X_{i}|Pa_{c}(X_{i})=u,Pa_{d}(X_{i})=j\right)=N(m_{j}+\beta_{j}\text{. }u,\;\sigma^{2}),\\ \;\left(m_{j},\;\beta_{j}|\sigma_{j}\right)\sim N(\mu_{j},\;\sigma_{j}\tau_{j}^{-1}),\\ \;\sigma_{j}\sim I\Gamma \left(\frac{\rho_{j}}{2},\frac{\phi_{j}}{2}\right). \end{array}$$

Given a network structure, the likelihood function and network score can be found in Bøttcher and Dethlefsen (2003, pp. 3–6, 11–12). We follow Bøttcher and Dethlefsen's implementation of Bayesian networks and also refer the reader to these publications (Friedman et al., 2000; Bøttcher and Dethlefsen, 2003; Bøttcher, 2004) for a complete discussion of Bayesian networks and the software (Bøttcher and Dethlefsen, 2003) we used to fit and analyze the data.

#### 2.1.1. Ranking network structures

All else equal, the best fitting network model can be identified by maximizing the log posterior probability of the network *G* given the data *d*, herein called the *network score* and denoted

(1) S(G)=logP(G|d)∝logP(d|G)+logP(G).

**Figure 1:**
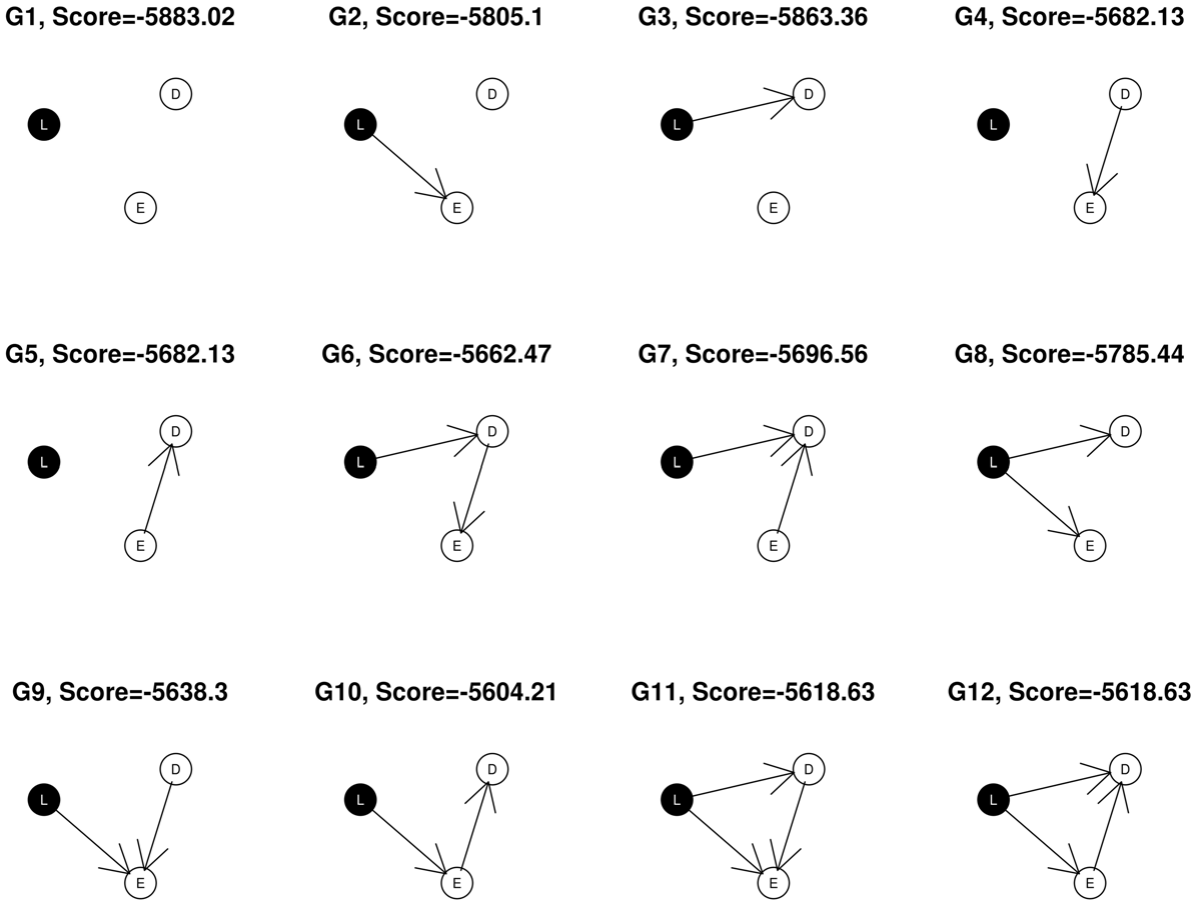
**All 12 possible networks for a given (*L, E, D*) triplet**.

In the simple example shown in Figure 1, nodes corresponding to SNP markers are denoted by *L*, expression by *E* and the disease, or phenotypic outcome by *D*. The SNPs, being discrete variables, are shown with a black background in the graphical network representation while the continuous nodes are shown in white. Assuming that gene expression level or phenotypic status could not change SNP genotypes, we restrict the possible network structures so that no edges come from the expression and phenotypic nodes to the SNP node at locus *L*, leaving a total of 12 possible DAGs that can be generated from the triplet {*L*, *E*, *D*}. In this example, the best fitting network structure for the {*L*, *E*, *D*} triplet will turn out to be *G*_10_ with *S* = −5558.75.

We have made a few adaptions to the likelihood-based network score *S*(G) to address certain practical concerns. When comparing network structures with different sets of nodes, and especially different numbers of nodes, the network scores of Equation (1) may be on different scales. And, all else equal, we prefer a network in which molecular variables are strongly associated with the phenotype *D* over one with very tight molecular associations but weak correlation with outcome. To achieve these goals, we define the average network improvement score (φ):

(2) $$\varphi =\frac{\lambda (S-S_{0})+(1-\lambda )S}{\alpha }.$$

where S is the network score of the structure under consideration, and *S*_0_ is the network score of its corresponding *null* network, obtained by removing the edge(s) to “*D*”. For example, for network structure *G*_10_ in Figure 1, the null network is *G*_2_. In addition, λ is a tuning parameter between 0 and 1, and α is the number of nodes considered in the network.

The quantity (S-S0) measures the improvement in the network score resulting from adding an edge to “*D*”. The numerator of φ is a weighted average of these two parts: the difference (*S* − *S*_0_) and the network score *S*. In addition, the tuning parameter, λ is used to adjust the weight of the two parts. To weight the two parts equally, we set λ = 0.5 in the following analysis.

To adjust for the number of nodes in network scores, we divide the numerator of φ by the number of nodes. This is used as a simple approximation of the effect of the number of nodes in the marginal likelihood. From Equation (1), when considering networks with no edges and assuming the nodes have the same distribution, the log marginal likelihood decreases linearly when adding nodes in the model, providing a heuristic justification for our specification.

The φ score so defined favors network structures that have both high posterior support and strong association with the phenotypic outcome. Using the previous example, the network score of *G*_10_ is −5558.75, and the score of its corresponding null graph, *G*_2_ is −5757.38. Hence, $\varphi =\frac{0.5[-5558.75-(-5757.38)]+(0.5\times -5558.75)}{3}=-893.35$.

### 2.2. Building networks

Our motivating hypothesis is that a network built on genomic sites and transcripts shown to be important in smaller network structures will be both accurate and computationally efficient. Accordingly we took a triplet—a SNP genotype taken together with an expression measure, and the phenotypic outcome—to be the basic building “module” in nPARS with larger networks formed by merging candidate triplets. The process can be divided into three main steps: (1) construct and score all triplets, (2) select the most informative of the resulting subnetworks, and (3) assemble these into larger networks. We will describe each of these in a little more detail in the next paragraph.

#### 2.2.1. Constructing three-node subnetworks

To form the basic building blocks, we decompose the whole network space into all possible (*L*, *E*, *D*) triplets, calculating a network score and best fitting structure for each. For the data set described in section 4, there are a total of 2330 × 3554 = 8,280,820 (*L*, *E*, *D*) triplets, and for each we find the network structures with the best network scores, as described above.

#### 2.2.2. Selection

Triplets are selected on the basis of the biological relevance of their best fitting network structures as well as the network scores. We exclude any (*L*, *E*, *D*) subnetworks containing a node of degree zero (having no connections with other nodes), so that only adequately connected networks are admitted for further analysis. Thus we select the subnetworks with structures shown as *G*_6_, *G*_7_, …, *G*_12_ in Figure 1. Next, we apply the average network improvement score (φ) to select the subnetworks that have both large support from the posterior and significant relevance to the outcome of interest. Subnetworks are ranked according to the φ scores. We then choose the top *k*_1_ subnetworks for further analysis. It is possible that after this step, there is only one (*L*, *E*, *D*) left. In this case, the algorithm reports this single three-node network. Our search in this step is exhaustive, which we find to be a significant strength of our approach.

#### 2.2.3. Reassembly

The final step is to build larger network structures from the chosen triplets. In doing this we considered first that the larger networks should contain two or more complete triplets, rather than mix and match individual nodes from different triplets, in order to preserve information that may be held jointly in those variables. Secondly, it should be permissible to reconstruct edges within triplets in addition to adding connections between triplets. These two considerations thus defined the assembly process, wherein a new Bayesian network is built *from scratch* using the nodes from a set of triplets. In our tests we assembled every pair of high scoring (*L*, *E*, *D*) triplets into four to five node networks, and used an exhaustive search to find the top scoring structure for each. We then build larger networks sequentially, adding additional triplets to the best scoring five node networks. At some point, as the networks get larger the exhaustive search option becomes computationally infeasible. This in fact happens fairly early, but we anticipate that the improved variable selection afforded by the modular approach would continue to pay dividends even if a greedy algorithm were used to construct edges at later stages in the assembly.

We summarize the three steps in the nPARS algorithm: construction of subnetworks, selection, and assembly as follows:

1. **Construction of subnetworks**:
  - Partition the whole network space into (*L*, *E*, *D*) subsets and
  - construct subnetworks.
2. **Selection**: Select the subnetworks with
  - network structures that are among *G*_6_, …, *G*_12_ in Figure 1 and
  - top *k*_1_ subnetworks with largest φ scores.
3. **Reassembly**:
  - Assemble two or more subnetworks into the union of their nodes;
  - Re-construct the assembled networks by scoring all possible network structures with the set of nodes.
  - Report the top *k*_2_ networks with largest φ scores.

**Figure 2:**
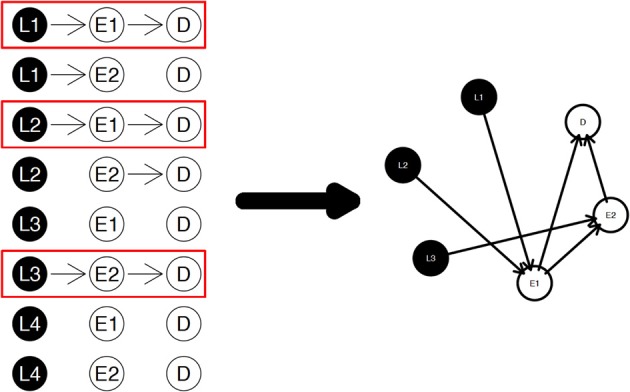
**The three steps in the nPARS algorithm: (1) constructing three-node subnetworks, (2) select subnetworks, and (3) reassemble into larger networks**.

The diagram shown in Figure 2 is a simple example for the nPARS algorithm described above. In Figure 2, eight triplet combinations are generated from four SNP loci (*L*_1_, *L*_2_, *L*_3_, and *L*_4_), and two expression measurements (*E*_1_ and *E*_2_). These three-node subnetworks are considered the basic building blocks (modules) of the nPARS algorithm. In the second selection step, three subnetworks are selected and a larger six-node network is re-constructed *from scratch* using the nodes from the selected subnetworks. In this example, *L*_4_ does not enter into the final reassembly step, since in the first step, the subnetworks associated with *L*_4_ do not connect with any expression (E) and phenotype (D).

## 3. Testing

To rigorously evaluate performance of our network partition and assembly approach (nPARS), we simulated a set of plausible gene networks, comparing our partition and assembly approach to an exhaustive (Exh) search on the one hand and a greedy search with random restarts on the other (Greedy). These algorithms are evaluated by comparing the reported final network structures to the assumed true network structure, to determine how frequently the correct nodes and edges are recovered. In these simulations we intentionally evaluate small networks, concentrating on the four- and five-node structures that result from joining two triplets. There are two reasons for this: (1) The exhaustive search for a best fitting network structure, which represents the gold standard of performance in these simulations, quickly becomes computationally prohibitive as a network gets larger. (2) We hope to model biologically plausible gene systems and to understand how features of those systems affect performance, and are not confident that human intuition is scalable in these regards.

### 3.1. Simulation settings

**Figure 3:**
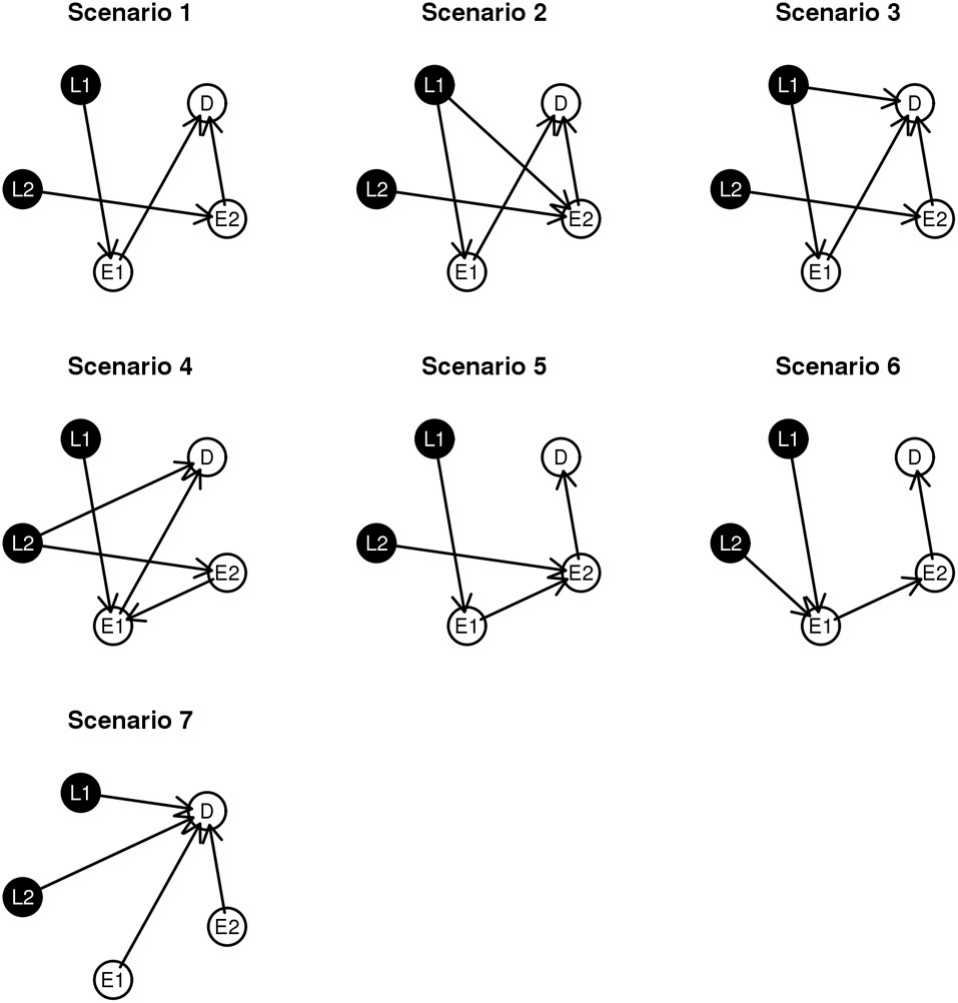
**Graphical representation of the simulation scenarios**.

To examine performance, we investigate seven simulated network structures, shown in Figure 3. Some of these scenarios are observed during the experimental data analysis presented in section 4 and others are developed from biological theory. For example, scenarios 1 and 2 are constructed based on the fundamental dogma of gene expression: DNA → RNA → phenotype. In scenario 3, 4, and 7, we add direct edges from *L* to *D* in keeping with structures identified in the course of analyzing experimental data. In addition, in scenario 5 and 6, we examine network structures with long connections (*L* → *E*_1_ → *E*_2_ → *D*). Scenario 7 could be considered as the worse case scenario because SNP loci contribute directly to D without alteration gene expression levels.

When simulating data, in order to mimic real world situations, we add unrelated SNP markers and expression measures as noise. The simulated data sets contain five SNP markers, five expression measures, and one continuous disease outcome. We simulate the data in the following four sample sizes: 100, 200, 500, 1000. SNP markers are simulated to have genotypes aa, Aa, and AA, with probability 0.25, 0.5, 0.25, respectively. Gene expression values from independent transcripts are simulated as *N*(10, $\sqrt{3.6}$). Expression values (*E*_*i*_) with edge effect β, for example from *L*_*i*_ are generated using the linear regression model: *E_i_* = 8 + β · *L_i_* + ϵ_*i*_, ϵ_*i*_ ~ *N*(0, $\sqrt{3.6}$). Phenotypic outcomes (D) are then generated based on genotype, and expression values through another linear regression model.

Specifically, we generate the simulated data using the following models: in scenario 1 and 2, *D* = $\frac{\beta }{2}$ · *E*_1_ + $\frac{\beta }{2}$ · *E*_2_ + ϵ_*i*_; in scenario 3, *D* = β · *I*(*L*_1_ = 1) + β *I*(*L*_1_ = 2) + β · *E*_1_ + β · *E*_2_ + ϵ_*i*_; in scenario 4, *D* = β · *I*(*L*_1_ = 1) + β *I*(*L*_1_ = 2) + β · *E*_1_ + β · *E*_2_ + ϵ_*i*_; in scenario 5, *D* = $\frac{\beta }{2}$ · *E*_2_ + ϵ_*i*_; in scenario 6, *D* = 3 β · *E*_2_ + ϵ_*i*_, and in scenario 7, *D* = β · *I*(*L*_1_ = 1) + β *I*(*L*_1_ = 2) + β · *I*(*L*_2_ = 1) + β *I*(*L*_2_ = 2) + β · *E*_1_ + β · *E*_2_ + ϵ_*i*_, where *I* is the indicator function. In the above equations, all ϵ_*i*_ are generated from *N*(0, $\sqrt{3.6}$). We evaluate the performance of each of the three algorithms for various β values.

### 3.2. Comparison of node recovery

#### 3.2.1. Algorithms

Three algorithms are implemented in this simulation study: nPARS, Exh, and Greedy. We apply nPARS as described previously. Specifically, in the selection step we keep all the subnetworks with more than one edge. In the final assembly step, we report the top 1 scoring network structure.

For comparison, in Exh, we define the network space to be all network structures that can be generated by all possible {*L*_1_, *L*_2_, *E*_1_, *E*_2_, *D*} five-node combinations, and exhaustively score all of them reporting the network with the largest φ score. In the simulation, we perform greedy search with 10 random restarts, stopping when the network score converges or when the algorithm reaches 100 iterations.

#### 3.2.2. Evaluation

Our first aim in the simulation analysis is to investigate whether nPARS recovers the correct nodes. For each true five-node network structure, we categorize the “nodes” in the final reported network structure as true positive (tp), false positive (fp) or false negative (fn) and evaluate the recovery of nodes using Sensitivity = $\frac{\text{tp}}{\text{tp}+\text{fn}}$, and FDR = $\frac{\text{fp}}{\text{tp}+\text{fp}}$.

#### 3.2.3. Results

**Figure 4:**
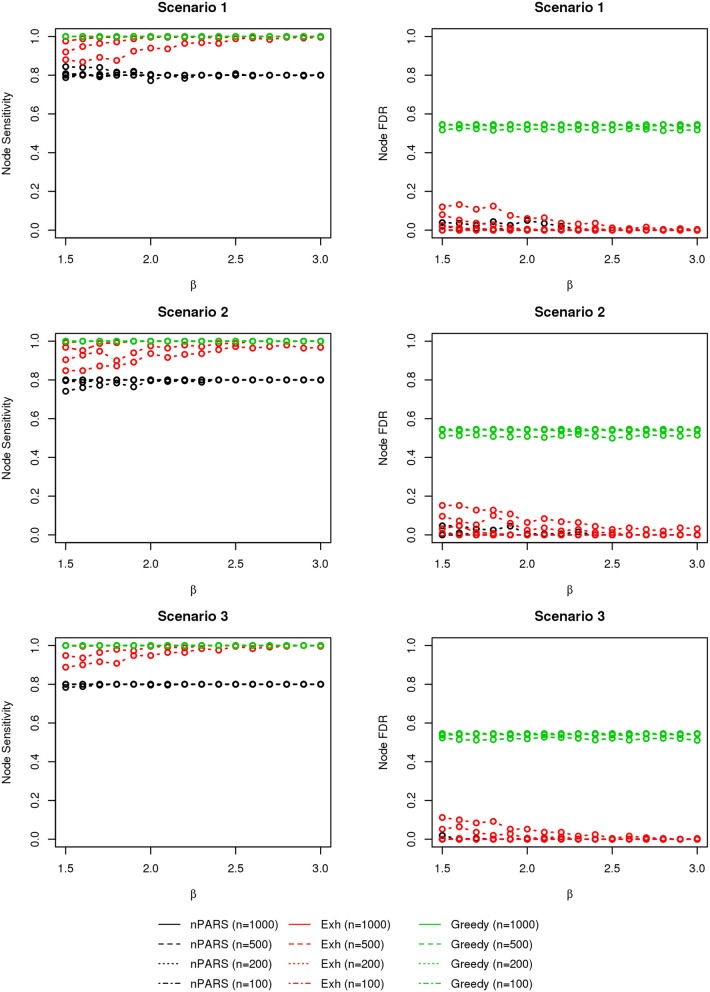
**The sensitivity and FDR of node comparisons in simulation scenario 1–3**. nPARS shows lowest node FDR with slightly lower node sensitivity compare to exhaustive search. Although greedy search exhibits highest node sensitivity but it reports relatively high node FDR.

**Figure 5:**
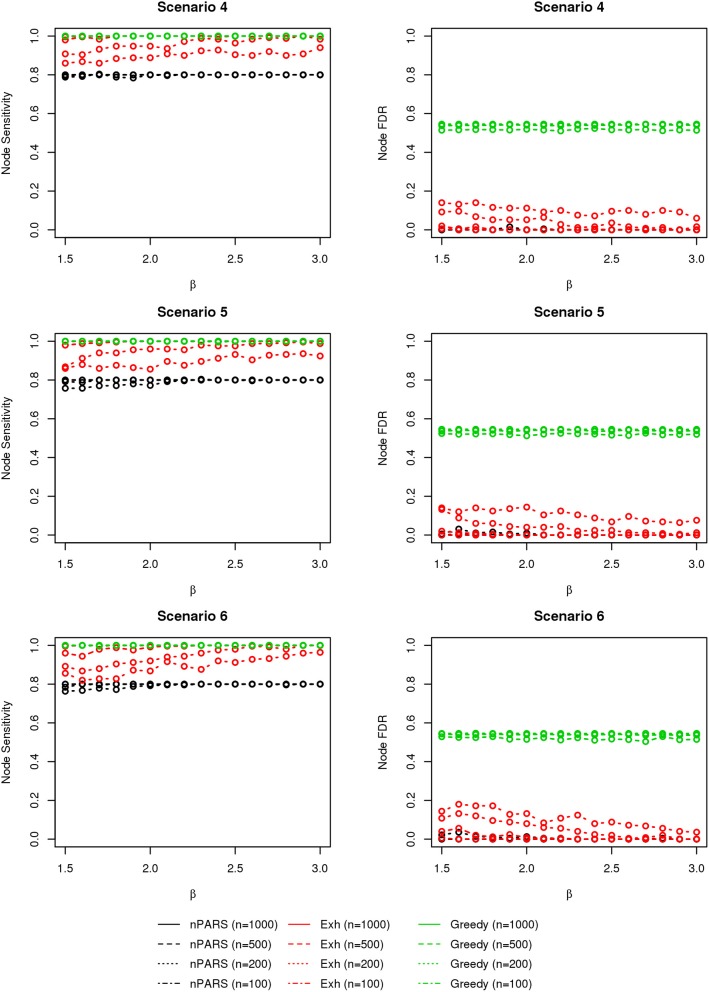
**The sensitivity and FDR of node comparisons in simulation scenario 4–6**. The same legend is used as in Figure 3. nPARS shows lowest node FDR with slightly lower node sensitivity compare to exhaustive search. Although greedy search exhibits highest node sensitivity but it reports relatively high node FDR.

**Figure 6:**
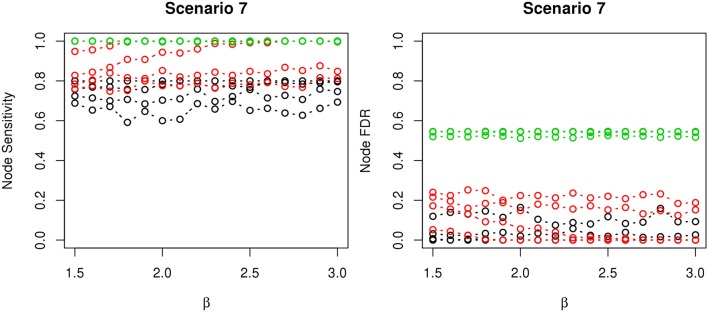
**The sensitivity and FDR of node comparisons in simulation scenario 7**. The same legend is used as in Figure 3. nPARS shows lowest node FDR with slightly lower node sensitivity compare to exhaustive search. Although greedy search exhibits highest node sensitivity but it reports relatively high node FDR.

The comparisons of node recovery are shown in Figures 4–6. In all simulation scenarios, nPARS (black line) exhibits slightly lower node sensitivity than Exh (red line) when sample size is the same. In addition, nPARS demonstrates lower node false discovery rate (FDR) than Exh and Greedy (green line) in all seven scenarios.

In addition, Greedy demonstrates highest sensitivity but also relatively large FDR in all simulation scenarios. In all simulation scenarios, Greedy reports networks with edge connections between almost all the nodes in the data. There are 6 out of 11 (54.5%) false nodes in the simulation dataset, and the average node FDR of Greedy search is 54.4% ($\frac{0.544}{0.545}$ ≈ 99.8%) when sample size (*n*) is less than 1000. In other words, Greedy falsely recovered 99.8% of the false nodes in the simulation dataset when *n* is less than 1000. This number decreases to 51.7% ($\frac{0.517}{0.545}$ ≈ 94.9%) when *n* = 1000.

### 3.3. Comparison of edge recovery

#### 3.3.1. Algorithms

The nPARS and Exh algorithms are implemented as described in section 3.2. However, as our findings from node recovery indicate, Greedy search often reports networks with too many nodes, and thus achieves high edge sensitivity at the price of a high number of false positive nodes. Hence, for edge the recovery comparison, it is desirable to control the number of nodes. In this analysis, we restrict the search space of the Greedy algorithm to network structures with no more than five nodes by adding an additional stopping rule requiring that when the network reaches five-nodes it stops. We call the revised version, GreedyE. As above, we categorize edges into tp, fp, fn, and calculate edge sensitivity and edge FDR based on the assumed true network.

#### 3.3.2. Results

**Figure 7:**
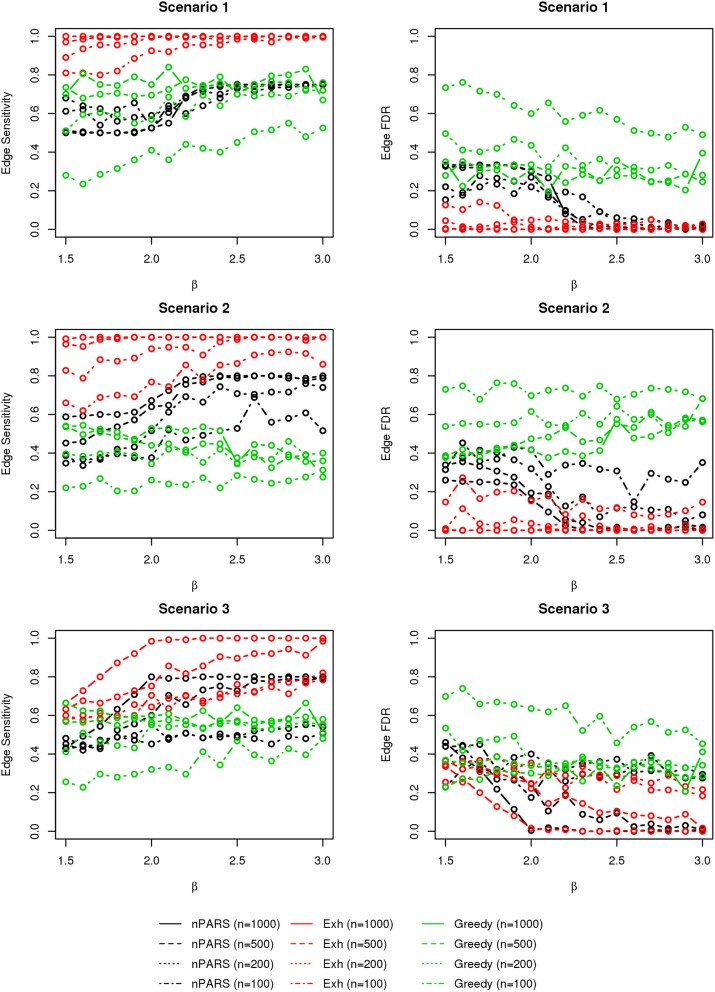
**The sensitivity and FDR of edge comparisons in simulation scenario 1–3**. nPARS has better performance than GreedyE in terms of edge sensitivity except in scenario 1 and 3. In scenario 1, nPARS has comparable edge sensitivity compare to GreedyE. nPARS has lower edge FDR compare to GreedyE.

**Figure 8:**
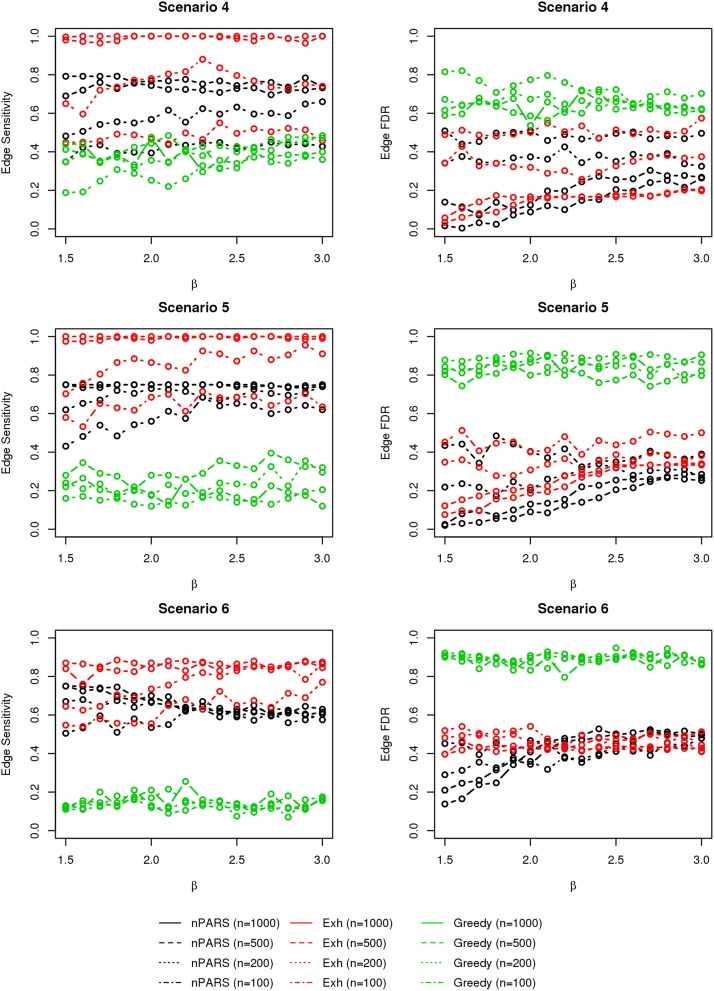
**The sensitivity and FDR of edge comparisons in simulation scenario 4–6**. The same legend is used as in Figure 6. nPARS has higher edge sensitivity and lower edge FDR compare to GreedyE in scenario 4–6.

**Figure 9:**
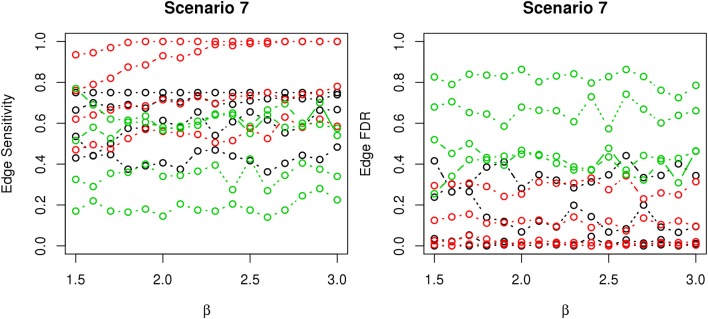
**The sensitivity and FDR of edge comparisons in simulation scenario 7**. The same legend is used as in Figure 6. nPARS has higher edge sensitivity and lower edge FDR than GreedyE in these scenarios.

In most of the studied scenarios, nPARS has better performance than GreedyE in terms of edge sensitivity, as shown in Figures 7–9, given the same sample size. The exceptions occur in a few instances in scenario 1, 3, and 7, when the edge effect β is small. When β is increased in scenarios 3 and 7, nPARS tends to have better edge sensitivity compared to GreedyE. In scenario 1, nPARS appears to have similar edge sensitivity compared to GreedyE. Exh has the best edge sensitivity recovery in almost all the scenarios.

In terms of edge FDR, GreedyE demonstrates the highest edge FDR in all simulation scenarios. nPARS shows similar edge FDR compare to Exh except in scenarios 1 and 2, when β is relatively small. In general, when considering both edge sensitivity and FDR, nPARS often demonstrates better edge sensitivity with the benefit of lower edge FDR compare to GreedyE. Exh has the best performance, however, in practice it is not feasible to implement Exh.

Overall, in the comparison with Greedy search, nPARS demonstrates lower FDR in both node and edge recoveries. In the comparison with Exh, nPARS demonstrates similar FDR in both node and edges recoveries but lower sensitivity. It is also notable, however, that nPARS achieved strikingly low, node FDRs in our tests, suggesting that the stepwise approach to network development may offer protection against over-fitting. For example, the stage 1 of nPARS requires that each expression node demonstrate a clear and simple link between some locus *L* and disease *D*, which makes it difficult for false nodes to make it to a full, five-node network in stage 2. In comparison, it could be relatively easy for the exhaustive procedure to complete a strong, four-node network with a noisy false fifth variable.

With regard to computational efficiency, under simulation scenario 1 with β = 0.8, nPARS takes about 32 s to complete 1 iteration, Greedy search takes about 52 s and Exh takes 2387 s (39 min and 47 s) with a single 2.3 GHz CPU core on a 64-bit AMD Opteron-based server. The run times are similar in magnitude under other scenarios. The time complexity of the nPARS algorithm depends on the parameters *k*_1_ and *k*_2_. The time complexity of the first step of nPARS grows linearly with increasing number of genes. If *k*_1_ and *k*_2_ are fixed regardless of the number of genes considered in the study, then the time complexity of nPARS algorithm grows linearly with increasing number of genes. The R source code and documentation of the nPARS algorithm are available at http://www.biostat.umn.edu/~yho/research.html.

## 4. Implementation

### 4.1. Cellular response to anticancer drugs data

In this example, we investigate differential responses to two chemotherapeutic agents: docetaxel and 5-FU. Both are widely used for a broad spectrum of cancers including colorectal, gastric, and head and neck cancer (Herbst and Khuri, 2003; Wang et al., 2004). Inter-individual variations in response to these anti-neoplastic drugs are commonly observed in cancer patients. Although several studies have shown that the resistance to docetaxel and 5-FU in human cancer cell are significantly inheritable (Watters et al., 2004), little is known about the underlying genetic mechanisms for this resistance.

This dataset includes 140 participants from 12 three-generation CEPH Utah families provided by the Genetic Analysis Workshop 15 (GAW15) (Cheung et al., 2005) and PharmGKB (Klein et al., 2001). Each family has approximately eight sibships in the third-generation. For each individual in the study, data from multiple sources was combined, including genotype, mRNA abundance, and cellular cytotoxicity levels in lymphoblastoid cells.

Genotypes of 2882 autosomal and X-linked SNPS, from across the whole genome, were generated by the SNP Consortium (http://snp.cshl.org/linkage_maps/) and provided through GAW15. We remove 552 SNP markers that have a high proportion of missing values (>0.3) or which are insufficiently polymorphic (minor allele frequency <0.1). We also examine the Mendelian consistency of the SNP genotypes and corrected them using Pedcheck and Merlin algorithms (O'Connell and Weeks, 1998; Abecasis et al., 2002).

Lymphoblastoid cells were isolated from each patient and 8793 mRNA transcripts were measured using Affymetrix Human Focus Arrays in previous studies (Cheung et al., 2003, 2005; Morley et al., 2004). We obtained the Affymetrix CEL files for all array hybridizations through GAW15. We then preprocessed the expression measures using RMA (Irizarry et al., 2003) and used mean expression intensities for replicates. For 3554 of the 8793 genes tested, Morley et al. (2004) found greater variation among individuals than between replicate determinations on the same individual. Hence, we choose these 3554 expression measures for further analyses.

The docetaxel and 5-FU cytotoxicity measures were obtained using lymphoblastoid cell lines derived from each participants and are available from the PharmGKB website http://www.pharmgkb.org/index.jsp. The percentages of LCL cell viability at 0.1, 0.5, 1, 5, 10, 50, 100 nM for docetaxel and at 0.76, 1.92, 3.84, 5.77, 7.68, 19.2, 38.4, 76.8 mM for 5-FU were measured and recorded for each individual. More detail about the cytotoxicity experiment procedures can be found in Watters et al. (2004).

### 4.2. Familial aggregation of responses to chemotherapeutic agents

**Figure 10:**
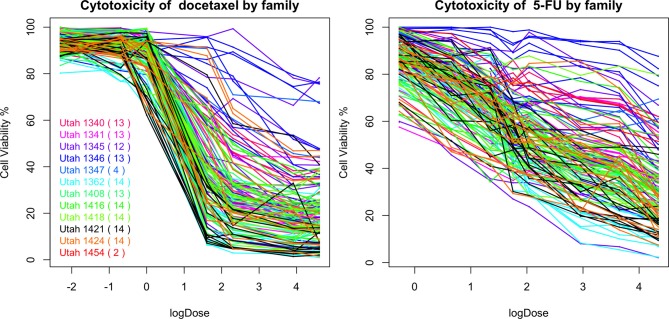
**The cytotoxicity responses of docetaxel and 5-FU**.

In Figure 10, we plot the percentages of cell viability against the log_*e*_ dose of docetaxel and 5-FU for each individuals. A large area under the log dose response curve indicates strong chemo-resistance. In the following analysis, for each individual, we use the area under the log-dose response curve as a summary representing the chemo-resistance outcome. There is one missing observation at 0.1 nM for docetaxel, there are four missing observations at 0.76 mM for 5-FU and there are no missing observation at the end dose for either agents. Since missing the first dose will underestimate the area under the curve, we apply linear regression models to predict the missing cytotoxicity values from non-missing observations at other does using data from the same individual.

Familial aggregation of the responses to chemotherapeutic agents can be observed. For example, individuals in the Utah 1346 pedigree (blue) show generally higher level of resistance than individuals in Utah 1424 (orange), Utah 1416 (green), and Utah 1362 (light blue) families in both graphs.

### 4.3. Results using nPARS algorithm

We apply the nPARS algorithm to this data, with 2330 SNP loci (*L*) and 3554 gene expression measures (*E*). We use the area under the log dose response curve as the phenotypic outcome (D), and analyze docetaxel and 5-FU separately. For each phenotypic outcome, we exhaustively score all possible 2330 × 3554 = 8, 280, 820 triplets combinations in the partition step. The subnetwork for each triplet is determined by the highest network score. Among these triplets, there are 825,637 (≈10.0%) triplets whose best fitted subnetworks are among *G*_6_, …, *G*_12_ for docetaxel and 635,390 (≈7.7%) for 5-FU.

**Figure 11:**
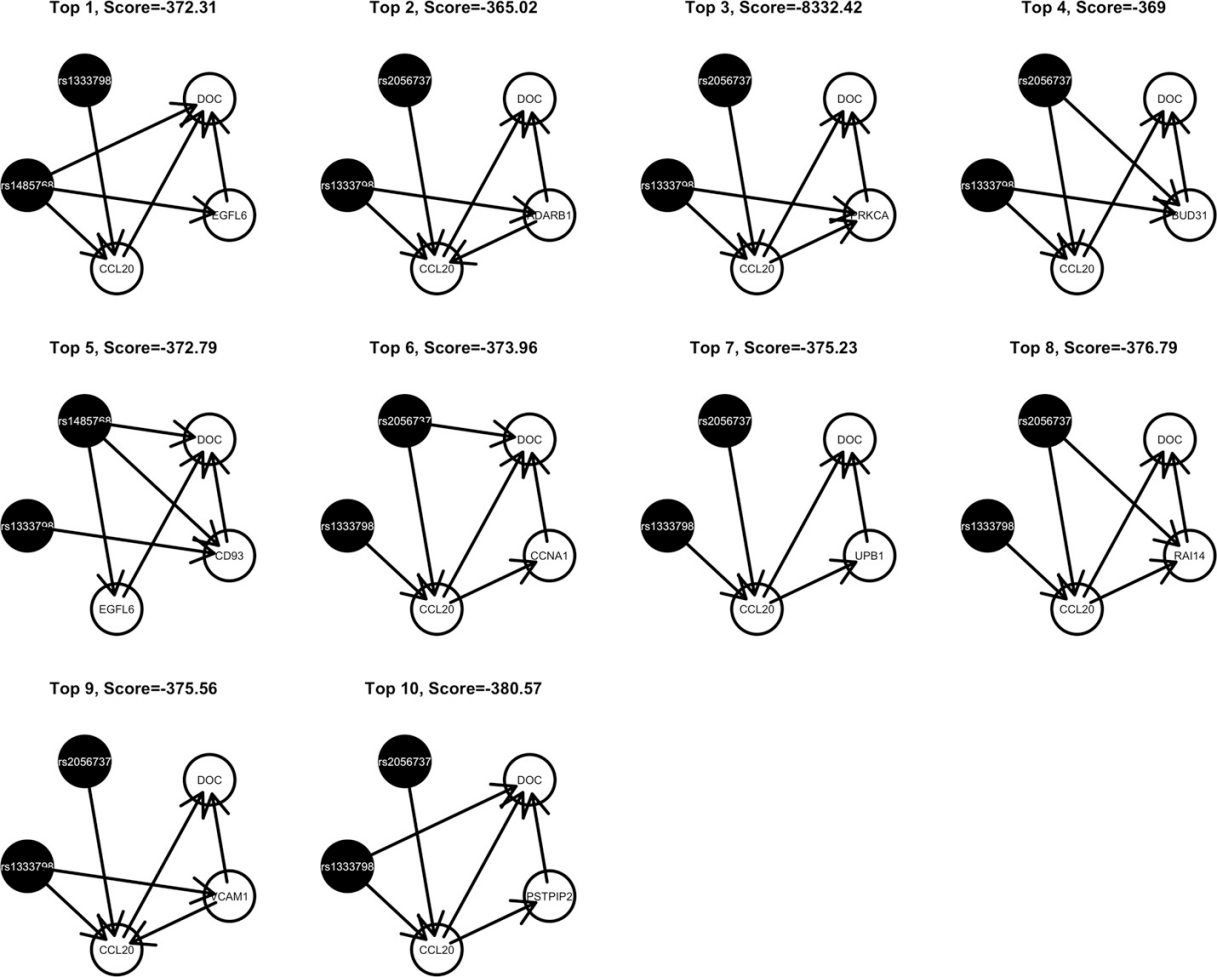
**Top scoring five-node network structures for docetaxel reported from nPARS algorithm**.

**Figure 12:**
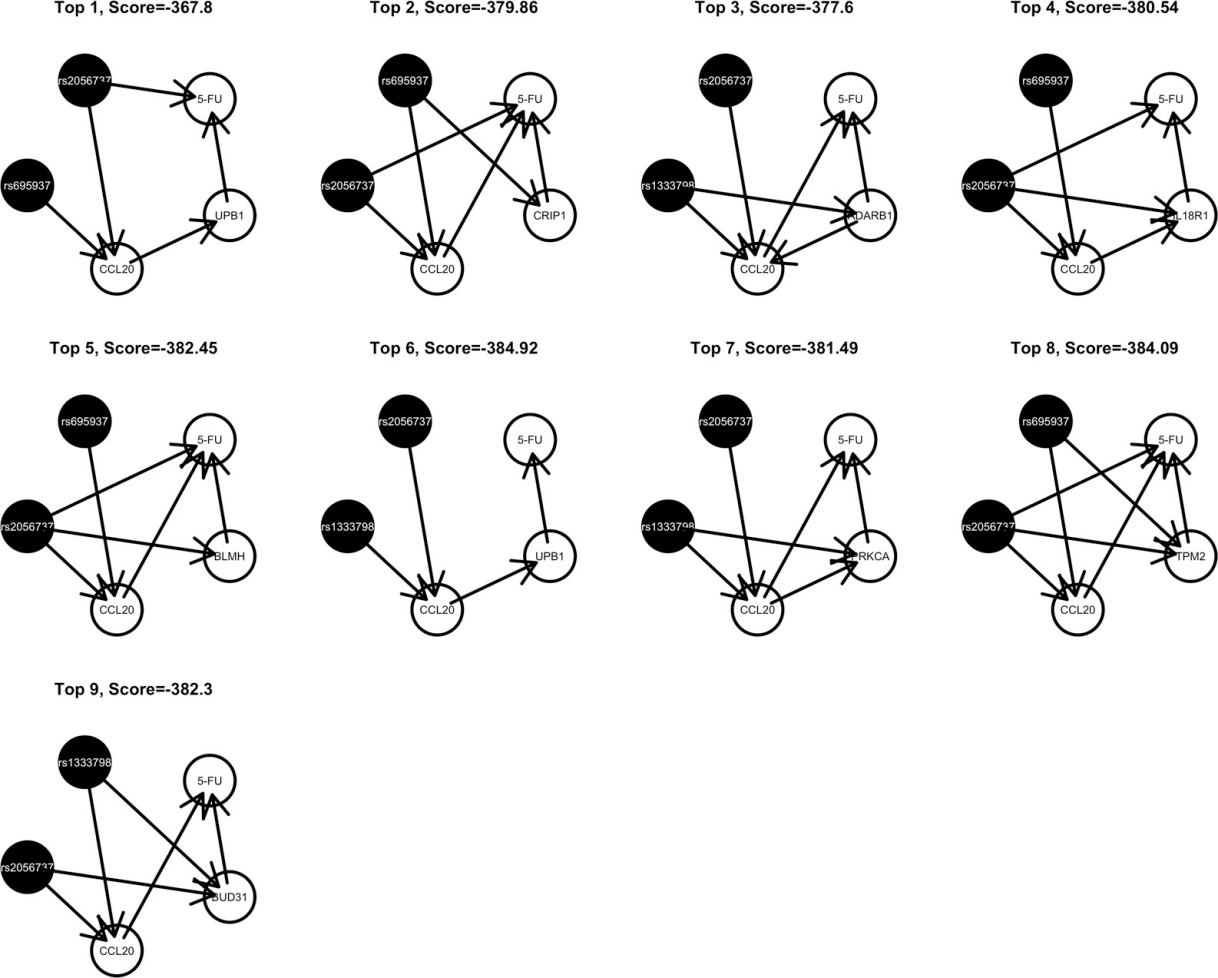
**Top scoring five-node network structures for 5-FU reported from nPARS algorithm**.

Among these, we select the top 100 scoring triplets for reassembly. We list the top 10 scoring triplets in Tables 1, 2 for docetaxel and 5-FU, respectively. Particularly, our results suggest four important SNP markers: rs1333798, rs695937, rs2056737, and rs1485768 because they appear many times in the top ranking networks for both docetaxel and 5-FU. In the subsequent reassembly step, we combine every two triplets into (100, 2) = 4950 sets of four or five nodes. After calculating the φ score for all resulting 4950 networks, we select the top 20. We present the five-node networks, if they have two gene expression as intermediate variables, in Tables 3, 4, for docetaxel and 5-FU, respectively. The corresponding network structures are plotted in Figures 11, 12.

**Table 1:** **Top 10 scoring triplets for docetaxel, and associated φ scores**.

	***L***	**Location of *L* (Chr: Mb)**	***E***	**Location of *E* (Chr: Mb)**	**φ**
1	rs1333798	13:88.8	CCL20	02:228.7	−103.04
2	rs695937	03:64.2	CCL20	02:228.7	−106.51
3	rs2056737	02:156.8	CCL20	02:228.7	−110.01
4	rs1333798	13:88.8	PSTPIP2	18:43.6	−113.34
5	rs1333798	13:88.8	SPARC	05:151.1	−113.64
6	rs1333798	13:88.8	PON2	7:95.1	−114.70
7	rs1333798	13:88.8	BUD31	7:99.0	−114.71
8	rs1485768	04:177.6	EGFL6	X:13.6	−114.81
9	rs1333798	13:88.8	VCAM1	01:101.2	−114.93
10	rs1333798	13:88.8	USP39	02:85.9	−115.56

**Table 2:** **Top 10 scoring triplets for 5-FU, and associated φ scores**.

	***L***	**Location of *L* (Chr:Mb)**	***E***	**Location of *E* (Chr:Mb)**	**φ**
1	rs695937	03:64.2	CCL20	02:228.7	−105.80
2	rs1333798	13:88.8	CCL20	02:228.7	−106.64
3	rs2056737	02:156.8	CCL20	02:228.7	−111.19
4	rs1333798	13:88.8	PON2	7:95.1	−114.17
5	rs1333798	13:88.8	FFAR2	19:35.9	−114.56
6	rs1485768	04:177.6	EGFL6	X:13.6	−114.71
7	rs1333798	13:88.8	UPB1	22:24.9	−115.30
8	rs2056737	02:156.8	FKBP5	6:35.6	−115.94
9	rs1015453	X:14.0	C5AR1	19:47.8	−115.98
10	rs2056737	02:156.8	TPM2	9:35.7	−116.62

**Table 3:** Top scoring five-node subnetworks for docetaxel^*^.

	***L*_1_**	***L*_2_**	***E*_1_**	***E*_2_**	**φ**	**Adjusted *R*^2^ (%)**
1	rs1333798	rs1485768	CCL20	EGFL6	−71.26	48.75
2	rs2056737	rs1333798	CCL20	ADARB1	−71.28	50.15
3	rs2056737	rs1333798	CCL20	PRKCA	−71.85	49.36
4	rs2056737	rs1333798	CCL20	BUD31	−71.96	47.96
5	rs1485768	rs1333798	EGFL6	CD93	−71.97	41.47
6	rs2056737	rs1333798	CCL20	CCNA1	−72.24	47.97
7	rs2056737	rs1333798	CCL20	UPB1	−73.23	50.09
8	rs2056737	rs1333798	CCL20	RAI14	−73.33	50.18
9	rs2056737	rs1333798	CCL20	VCAM1	−73.37	48.96
10	rs2056737	rs1333798	CCL20	PSTPIP2	−73.4	51.36

*All p values <0.00001*.

**Table 4:** **Top scoring five-node subnetworks for 5-FU**.

	***L*_1_**	***L*_2_**	***E*_1_**	***E*_2_**	**φ**	***p* value**	**Adjusted *R*^2^ (%)**
1	rs2056737	rs695937	CCL20	UPB1	−71.46	3.81×10^−5^	40.51
2	rs695937	rs2056737	CCL20	CRIP1	−74.24	1.03×10^−4^	37.85
3	rs2056737	rs1333798	CCL20	ADARB1	−74.62	3.38×10^−3^	25.72
4	rs695937	rs2056737	CCL20	IL18R1	−74.69	1.07 ×10^−5^	43.69
5	rs695937	rs2056737	CCL20	BLMH	−75.09	3.40 ×10^−5^	40.81
6	rs2056737	rs1333798	CCL20	UPB1	−75.41	1.13 ×10^−3^	29.41
7	rs2056737	rs1333798	CCL20	PRKCA	−75.46	6.80 ×10^−3^	23.17
8	rs695937	rs2056737	CCL20	TPM2	−75.52	3.57×10^−4^	34.24
9	rs1333798	rs2056737	CCL20	BUD31	−75.59	0.01	21.68

To estimate the variance explained by the top scoring networks, we perform linear regression adjusting for family. In the regression model, we used the area under the log-dose response curve as response variable and the nodes reported in the final networks (shown in Tables 3, 4) as predictors, while adjusting for family. The results are shown in the final column in Tables 3, 4 for docetaxel and 5-FU, respectively. After adjusting for family structure, we observe that the top scoring networks reported by nPARS explain a significant amount of variation in drug resistance outcomes. The mean adjusted *R*^2^ are 48.63% and 33.01% for docetaxel and 5-FU, respectively. In addition, we obtain *p*-values using an F test based on linear regression models. All top scoring networks show *p*-value smaller than 0.00001 for docetaxel and smaller than 0.01 for 5-FU. Even after Bonferroni correction for multiple comparisons, all remain statistically significant except subnetwork #7 and #9 for 5-FU.

Through this experimental data analysis, we intend to demonstrate the implementation of nPARS in a large-scale genomic data set. The analysis results suggest that rs1333798, rs1485768, rs2056737, and rs695937 and CCL20 combinations might explain the cytotoxicity responses observed in the lymphoblastoid cell lines for both docetaxel and 5-FU. rs1485768 is within the VEGFC gene which is involved in multiple cancer related pathways. In addition, rs695937 locates within the PRICKLE2 gene coding region. PRICKLE2 belongs to the Wnt signalling pathway which regulates many downstream genes through its interaction with the T-cell factor family of transcription factors. The wnt signaling pathway also leads to remodeling of the cytoskeleton which is the main drug action of docetaxel, though the exact connection between these genetic variants and CCL20 expression is not yet clear.

CCL20 is a chemokine and it provokes proliferation and adhesion to collagen for several types of cancer cells (Beider et al., 2009). It is also believed that CCL20 is relevant to chemo-resistance for various kind of cancers (Chang et al., 2008). For docetaxel resistance in lymphoblastoid cells, it is possible that CCL20 may influence resistance through regulation of actin cytoskeleton via the chemokine singling pathway, since cytoskeleton function is the main drug target of docetaxel. Genes' expressions that are likely to co-regulate with CCL20 and contribute to docetaxel resistance include EGFL6, ADARB1, PRKCA, BUD31, CD93, CCNA1, UPB1, RAI14, VCAM1, and PSTPIP2. Some of these genes are likely to be relevant to chemo-resistance response through cell cycle regulation, adhesion, or carcinogenesis pathways, EGFL6, PRKCA, VCAM1. ADARB1 and BUD31 are involved in mRNA precursor editing and modification. CD93, RAI14, and PSTPIP2 are part of cytoskeleton or interact with cytoskeleton function.

In addition, as indicated in the reported top fifth scoring network, the genetic variations in two SNP markers: rs1485768 and rs1333798 might contribute to the variation in gene expression of EGFL6, CD93. EGFL5 and CD93 playing important roles in regulating cell cycle, and remodeling cytoskeleton.

As for resistance to 5-FU, the CCL20 chemokine is also found to be crucial. CCL20 might play an important role through mediating DNA degradation or GPCR pathways. Other genes that could potentially co-regulate 5-FU resistance together with CCL20 include UPB1, CRIP1, ADARB1, IL18R1, BLMH, PRKCA, TPM2, BUD31, ITGAM, and RAB8B. Specifically, UPB1 participates in the 5-FU drug metabolic pathway by converting fluoro-beta-ureidopropionate to fluoro-beta-alanine (FBAL). FBAL is the major secretable form of 5-FU found in patients' urine sample. Although feasible biological hypotheses could be suggested based on our analysis results, further experiments are needed to validate the roles of these genetic factors in chemotherapy response.

## 5. Conclusion

To meet the growing need for efficient data analysis at the level of biological systems, we have developed and evaluated a modular approach to the construction of genetic networks. Our goal was to strike an appropriate balance between two potential sources of error. There is the error introduced when a necessarily less-than-exhaustive search through high-dimensional network space misses important regions of that space. This risk can be reduced by judicious variable selection to reduce the size of the search, but “judicious” is a loaded term and ideally the variable selection step would capture some of the information that is distributed jointly across network components. By building a network from small components identified in an exhaustive search we hope to improve variable selection while controlling the computational burden.

The main focus of the paper is to assess the advantage of network-driven feature selection strategy. Based on our study findings, this network construction strategy provides ways to focus on small subnetworks that present with higher signal and allow more reliable estimation of network structure. In a set of extensive simulations, we compared the performance of the modular nPARS approach to that of both the greedy and exhaustive searches, evaluating the performance of each across a variety of scenarios. In these analyses, nPARS outperformed the greedy search which tended to have high FDRs for both nodes and edges, and proved competitive with the exhaustive search.

The fact that nPARS achieves better performance in terms of false discovery than exhaustive search in some simulation scenarios is beyond our expectation, and we suggest two possible factors: (1) Although we have attempted to represent a range of biologically realistic networks, there may be some bias in the system whereby the variable selection criteria implicit in nPARS is particularly appropriate to the network structures modeled in some of those scenarios. (2) One of the goals driving this method was to improve the effectiveness of the search through network space by including only those variables that made a significant contribution to smaller network structures. By requiring clear links between locus L, transcript E and phenotype D in the first stage of the algorithm, we make it less likely that a noisy false node is available for inclusion in the larger network later on. Without such a filtering step, it is relatively easy for the exhaustive procedure to complete a strong four-node network with a noisy, false fifth node. By either cause, we would anticipate that in larger, more complex networks, that nPARS' advantage over the exhaustive procedure would diminish. Unfortunately it is not yet practical to scale the exhaustive approach to test this.

We did not explicitly model family structure when constructing the Bayesian networks on our chemo-resistance application, assuming that any similarity of phenotypic values between relatives could be fully explained by the genetic variables considered in a network. However, since pedigree data was available for the samples in the drug response study, we used it in evaluating the top scoring networks we reported. Specifically, we performed a linear regression analysis that included family structure, to see how well the genetic variables explained drug response after adjusting for pedigree structure. We obtained small *p*-values and large adjusted *R*^2^, suggesting that the reported networks play significant roles in drug resistance responses.

Other limitation of the proposed nPARS algorithm is that the algorithm in its current specification focuses on identifying structures related to (L, E, D). As demonstrate by simulation scenario 7, nPARS has considerable power to detect cases where L contribute to D directly (*L* → *D*). However, in scenario 7, if we replace *E*_1_ and *E*_2_ by *L*_3_ and *L*_4_, then nPARS would have a diminished power to detect such case. The algorithm can be easily modified to consider this modified scenario but increased amount of computational intensity will be expected.

Furthermore, our implementation of nPARS is tailored to the SNP—expression—phenotype setting in which it was tested, but could be readily modified to accommodate other genetic or epigenetic data in place of SNPs, including copy number and DNA methylation, though it may be necessary to modify the scoring functions or re-weight the prior distribution on network structures to reflect the unique biological characteristics of each data type. Potential direction for future research is to accommodate pedigree structure into the marginal likelihood score of Bayesian networks. But this approach would require considerable amount of samples to have enough power for detecting effects. We anticipate to have demonstrated that a practical compromise between exhaustive and greedy searches can improve on both and that our method can be the basis for future expansions.

### Conflict of interest statement

The authors declare that the research was conducted in the absence of any commercial or financial relationships that could be construed as a potential conflict of interest.
